# Biomonitoring as an Underused Exposure Assessment Tool in Occupational Safety and Health Context—Challenges and Way Forward

**DOI:** 10.3390/ijerph17165884

**Published:** 2020-08-13

**Authors:** Susana Viegas, Maryam Zare Jeddi, Nancy B. Hopf, Jos Bessems, Nicole Palmen, Karen S. Galea, Kate Jones, Peter Kujath, Radu-Corneliu Duca, Hans Verhagen, Tiina Santonen, Robert Pasanen-Kase

**Affiliations:** 1 NOVA National School of Public Health, Public Health Research Centre, Universidade NOVA de Lisboa, 1600–560 Lisbon, Portugal; 2 Comprehensive Health Research Center (CHRC), 1169–056 Lisbon, Portugal; 3 H&TRC—Health & Technology Research Center, ESTeSL—Escola Superior de Tecnologia da Saúde, Instituto Politécnico de Lisboa, 1500–310 Lisboa, Portugal; 4 Unit of Biostatistics, Epidemiology and Public Health, Department of Cardio-Thoraco-Vascular Sciences and Public Health, 35100 Padova, Italy; maryam.zarejeddi@unipd.it; 5 Center for Primary Care and Public Health (Unisanté), University of Lausanne, 1000 Lausanne, Switzerland; nancy.hopf@unisante.ch; 6 VITO—Flemish Institute for Technological Research, BE-2400 Mol, Belgium; jos.bessems@vito.be; 7 RIVM—National Institute for Public Health and the Environment, 3721 MA Bilthoven, The Netherlands; nicole.palmen@rivm.nl; 8 Institute of Occupational Medicine (IOM), Edinburgh EH14 4AP, UK; karen.galea@iom-world.org; 9 Health and Safety Executive (HSE), Harpur Hill, Buxton SK17 9JN, UK; kate.jones@hse.gov.uk; 10 BAuA—Federal Institute for Occupational Safety and Health, D-10317 Berlin, Germany; kujath.peter@baua.bund.de; 11 Unit Environmental Hygiene and Human Biological Monitoring, Department of Health Protection, National Health Laboratory, Dudelange, 3555 Luxembourg, Luxembourg; radu.duca@lns.etat.lu; 12 Centre Environment and Health, Department of Public Health and Primary Care, KU Leuven, 03000 Flanders, Belgium; 13 European Food Safety Authority (EFSA), 43126 Parma, Italy; Hans.Verhagen@efsa.europa.eu; 14 Nutrition Innovation Center for Food and Health (NICHE), University of Ulster, Coleraine BT52 1SA, UK; 15 FIOH—Finnish Institute of Occupational Health, P.O. Box 40, FI-00032 Työterveyslaitos, Finland; tiina.santonen@ttl.fi; 16 State Secretariat for Economic Affairs (SECO), Labour Directorate Section Chemicals and Work (ABCH), 3003 Berne, Switzerland; robert.pasanen-kase@seco.admin.ch

**Keywords:** biological monitoring, exposure assessment, risk assessment, occupational health, biological guidance value, biological limit value

## Abstract

Recent advances in analytical chemistry have allowed a greater possibility of using quantitative approaches for measuring human exposure to chemicals. One of these approaches is biomonitoring (BM), which provides unequivocal evidence that both exposure and uptake of a chemical have taken place. BM has been a longstanding practice in occupational health for several reasons. BM integrates exposure from all routes. It can help identify unintentional and unexpected exposures and assess the effectiveness of existing risk-management measures. BM also provides relevant information to support policy development by delivering better evidence of workers’ exposure to chemical substances, even within the framework of the present regulations. Thus, BM can allow for both the evaluation of the impact of regulation and identification of further needs for new or improved regulation. However, despite all these well-recognized advantages, BM is currently an underused exposure assessment tool. This paper provides an overview of the key aspects to be considered when using BM in the context of occupational health interventions. Additionally, this paper describes the potential of BM as an exposure assessment tool, distinguishing the role of BM in exposure assessment and health surveillance and clarifies ethical and communication aspects to guarantee that general data protection regulations are followed. In addition, actions and research needs are identified (particularly with reference to the European situation), which aim to encourage the increased use of BM as an exposure assessment tool.

## 1. Introduction

Exposure assessment is defined as the process of estimating or measuring the magnitude, frequency and duration of exposure to an agent and the size and characteristics of the population exposed [1]. Biomonitoring (BM) is a quantitative approach for assessing exposure and recent advances in analytical chemistry have allowed for increased opportunities to use this approach.

BM measures chemicals and their metabolites in the human body, usually through analyses of blood, urine, hair, breast milk or exhaled breath condensate. It provides an aggregated measure of the level of exposure to chemicals through different exposure routes. BM is an important tool for assessing exposures to chemicals among the general population and workers [2,3].

Whilst occupational exposure to chemicals continues to be an important cause of mortality and health burden worldwide, the need for ongoing prevention and control initiatives is justified [4,5,6]. In occupational health interventions, BM can assist in the evaluation and demonstration of the effectiveness of policy actions, measures already taken at a company level to reduce exposure and the detection of emerging and well-known contaminants. Additionally, and since BM considers aggregated exposures (exposure to the same substance from different sources and by different exposure routes), it provides a comprehensive exposure picture compared to ambient monitoring (e.g., air monitoring, surfaces contamination measurement, skin contamination) [2,7].

Moreover, one of the main advantages of BM data is that they offer potential communication advantages over ambient monitoring data. For instance, Sexton et al. stated that they yield “unequivocal evidence that both exposure and uptake have taken place” ([8] p.41). As a result, BM data are often considered the most valid and persuasive evidence available to demonstrate whether, and to what extent, exposure has occurred or changed over time [9,10]. A study intending to assess exposure to polycyclic aromatic hydrocarbons of off-shore workers by using urinary 1-hydroxypyrene levels concluded that the worker with the greatest exposure was not the one performing the tasks but was another worker who was present for other reasons and thought that a respirator would not be needed [11]. BM was also useful for giving direct feedback to individual workers regarding their exposures in the electroplating industry where workers contributed significantly in reducing their exposures [12]. These are good examples of how BM can really make an impact demonstrating specific exposure differences even when the workers are present in the same workplace environment.

BM of occupational exposure to chemicals is a longstanding practice in occupational health risk assessment and occupational health practice [7]. However, there are wide differences in its use between different countries, as identified in a recent European Commission (EC) survey aimed at understanding BM usage in Europe [13].

In the occupational health context and according to Manno et al., BM could have three main goals: 1. individual or collective exposure assessment; 2. health protection; but also 3. occupational health risk assessment [2]. However, BM includes the collection of biological specimens from the workers, it is typically applied as a part of health surveillance programmes under the supervision of an occupational health professional. Consequently, in many countries, results are commonly considered as medical data for which strict confidentiality rules apply and, as such, are not available for use in workplace exposure and risk assessment (goals 1 and 3 according to Manno et al. [2]). This is reiterated by the fact that within the European occupational safety and health (OSH) legal framework (in CAD—the Chemical Agents Directive: Directive 98/24/EC—Risks related to chemical agents at work), BM is mentioned as only a health surveillance tool and not as an exposure assessment tool. However, in 1996, the World Health Organisation (WHO) had already defined BM and health surveillance as two different things: BM as “the measurement and assessment of workplace agents or their metabolites either in tissues, secreta, excreta, expired air or any combination of these to evaluate exposure and health risk compared to an appropriate reference” and health surveillance as “the periodic medico-physiological examinations of exposed workers with the objective of protecting health and preventing occupationally related disease” [14]. The WHO also mentions that exposure can be assessed by ambient monitoring or by biological monitoring [14].

In 1998, the International Labour Office defined occupational health surveillance as “the ongoing systematic collection, analysis, interpretation and dissemination of data for the purpose of prevention” [15]. According to the European Union (EU) CAD, health surveillance is statutory for specific chemical agents for which a binding biological limit value (BBLV) exists. Lead is the only substance that has a BBLV in the EU to date and it is set for blood lead (B-Pb), with a direct link to health effects. In the case of lead, BM helps to identify workers at possible risk for adverse health conditions. In such cases, an occupational physician will inform the employer whether the worker is fit to work or needs to change their workplace/working conditions. However, the occupational physicians cannot communicate the reason for the decision without the permission of the worker, as this is considered medical information. Limiting the use of BM under health surveillance is one of the main issues hampering the routine application of BM as an exposure assessment tool on a large scale. BM is useful for assessing exposure to a wide variety of chemicals, and has the potential to provide detailed data about exposure and guarantee an adequate risk assessment and management. This thinking evolved early on in the US where BM is used as an additional tool by industrial hygienists to aid in the assessment of worker exposures, belonging to the occupational hygiene field rather than health surveillance [16].

BM is a potentially important tool for OSH research, practice, and policy but the full extent of these tools has yet to be realized. Therefore, the focus of this paper is to describe the potential of BM as an exposure assessment tool in the context of occupational health, distinguish the role of BM in exposure assessment and health surveillance and, finally, to clarify ethical and communication aspects to increase the use of BM as an exposure assessment tool in the occupational health context. Although the paper covers these aspects in a generalised way, we also review the place of biomonitoring in European occupational safety and health regulations, in particular.

Considering the relevance of the subject for the exposure science community, this paper was developed with the support of the International Society for Exposure Science–European Chapter (ISES Europe), an interdisciplinary network of experts in different regulatory and research roles to further improve and reinforce the use of biomonitoring in occupational health.

## 2. Additional Information That Biomonitoring Provides When Assessing Occupational Exposure

The most common reasons to perform occupational exposure assessments are to evaluate compliance with legal requirements, to measure workers’ exposure and to evaluate the effectiveness of risk management measures (RMM). In all of these, BM can play a relevant role when, besides inhalation, other exposure routes (dermal absorption and ingestion due to hand-to-mouth contact) have an important contribution to the workers’ overall exposures. Thus, BM may be particularly useful for those chemicals that are easily absorbed through the skin or taken in by ingestion due to their permanence on workplace surfaces, or where exposure is controlled only by personal protective equipment (PPE) and thus is dependent on the PPE quality, correct selection and use [17].

BM therefore has advantages compared to the common practice of ambient monitoring because it provides additional data (it considers the exposure from all routes). In the case of substances with long half-lives that accumulate (e.g., perfluoroalkyl and polyfluoroalkyl substances (PFAS), cadmium, lead) BM reflects total exposure over time. In addition, BM aggregates exposures from all sources; at work, at home and from other environments (e.g., mycotoxins, phthalates) [18,19]. Thus, exposure to a substance that is regulated under different legislative frameworks (e.g., OSH and food safety) is also considered and analyzed. In addition, BM takes into account differences in metabolism between humans (polymorphism) since actual concentrations in body fluids are measured. It also takes into account the impact of physical activity on the inhaled volumes and total exposure, which is especially important in labour-intensive jobs. Interactions between different substances are also captured with BM data and they may play a role since workers are commonly exposed to mixtures or to different substances during the day. Interactions may lead to altered internal exposure of the parent compound or their metabolite(s) and even to different metabolism and excretion patterns [20,21]. As an example, in a survey developed in a polymer-producing facility where workers were exposed to either styrene alone, xylene alone or a combination of both, potential to induce genomic instability was observed either alone or in combination, showing higher effects when combined [22].

BM may also give insight into time-trends of total internal exposure of a substance where BM is performed on a regular basis. In this way it will give information on the effectiveness of RMMs already taken. In addition, stratification of similar exposure groups of workers can be based on BM data. This information can help occupational hygienists to determine whether and where exposure reduction measures would be most effective. For substances with high background levels of exposure (due to for example environmental contamination, lifestyle factors, diet, medication) BM data might need additional analysis for interpretation. Thus, with adequate contextual data (e.g., information about tasks, how tasks are developed, risk-management measures in place, and others) BM data are able to answer several questions related with risk assessment and management such as: What is the total exposure? What is the contribution of other exposure routes besides inhalation (e.g., dermal absorption, ingestion due to hand-mouth contact)? Are there opportunities to reduce exposures? These questions involve interpretation of BM data but also relate with risk management issues, implying the collection of contextual data (e.g., RMMs in place, tasks performed, how workers undertook the tasks, hygienic conditions of the workplace, worker behaviours such as eating in the workplace or not washing their hands before eating or smoking). As with ambient monitoring data, BM will also make workers aware of the need to work safely since they will be given individual BM values compared to the limit or guidance value in place [22].

Furthermore, BM can play a role in two different strategies: in a collective approach, to support the need to improve working conditions and to provide information for an exposure management strategy and, in a more individual approach, to give an answer related to health risk of each worker. At the individual level, monitoring results can lead to the introduction or improvement of exposure control measures and individual working practices within the working environment and, in some special circumstances (like in the case of high lead accumulation in the body), job replacement. In the collective method, monitoring data can be used to assess the effectiveness of exposure controls, develop risk communications, and identify failures of prevention and control. Therefore, it should be possible to analyse BM data on both levels to protect workers [23]. However, independent of the approach, we will need to consider and address the following questions: Why do we want to use BM? Is there a suitable (validated) biomarker (of exposure)? Is the BM tool being used to assess exposures or assess an individual worker’s health risk? Will the data bring additional value for the assessment of the contributions of different exposure routes? How will BM results help in identifying new needs concerning RMMs?

In epidemiological studies, exposure information is essential in investigating dose-response relationships between a chemical and a certain health effect. Indeed, the lack of insight in quantitative total exposure information of a substance is often a problem in epidemiological studies. When standardized guidelines become available for measuring many chemicals simultaneously and high throughput analyses exist, then identification of new dose-response relationships may be possible, linking chemical body burden to specific health outcomes. Certainly, BM may contribute significantly to further developing quantitative risk assessments [7].

To summarise, BM data can support several occupational health information needs, namely: providing knowledge of exposure by all exposure routes, determine if a specific exposure has occurred and if it implies a risk to health; helping practitioners to understand the results from clinical testing in some circumstances; recognising the adequacy of the RMMs in place; demonstrating the link between an occupational exposure and a health effect. Finally, BM data can support health monitoring and surveillance programmes, and identify possible trends in exposure [17,24,25,26].

## 3. Biomonitoring in Occupational Safety and Health Regulations

### 3.1. Biomonitoring Values to Aid Data Interpretation (Regulated, Advised and Reference Values)

There are several types of biological value in occupational settings (regulated or advised) with the following terminology in the European Union: biological limit values (BLVs) which are health-based, or biological guidance values (BGVs; also called reference values) which are not health-based but are statistically derived. BGVs indicate background internal exposures in the general population (or occupationally non-exposed population). Specifically, a BGV represents the concentration of the substance (or a metabolite) in any appropriate biological medium corresponding to a certain percentile (generally 90 or 95 percentile) in a defined reference population [7,27]. If background levels cannot be detected, the BGV may be equivalent to the detection limit of the biomonitoring method, which then is to be specified in the BGV documentation [28,29]. Health-based BLVs are usually derived by following approaches: (1) When appropriate epidemiological data are available to link adverse effects directly with biomarker levels, the no observed adverse effect level (NOAEL) may directly be used to derive the BLV. (2) If such studies are not available but an occupational exposure limit (OEL) has been set and studies in humans provide a link between airborne concentrations of the compound and concentrations of the compound or its metabolites in biological media, a BLV may be recommended that corresponds to the OEL. Supporting evidence may be drawn from toxicokinetic modelling. However, for substances for which the OEL is not established on the basis of systemic effects but because of local irritation, a BLV may still be based on systemic adverse effects. In such (exceptional) cases where the OEL and BLV are based on different end points, the two values may not necessarily correlate [30].

An overview of different types of occupational limit values is presented in Table 1. With regard to external exposure, a distinction is made between binding occupational exposure limit values (BOELVs) and indicative occupational exposure limit values (IOELVs). BOELVs are not necessarily health based and may be set higher than a health-based recommended limit value taking into account socio-economic and technical feasibility factors. Therefore, when setting a BOELV policy considerations are of major importance [31].

In order to permit an easier interpretation of BM results, biological limit or guidance values are required. A distinction is also made here between not necessarily health-based Binding Biological Limit Values (BBLVs), BLVs which are health-based threshold values, and BGV. At this moment there is only one BBLV in the European Union published in the CAD; which is set for lead and its ionic compounds. Member states are obliged to set a national BBLV that is lower or equal to the value published in the CAD. BLVs are currently only given as SCOEL (Scientific Committee on Occupational Exposure Limits) or RAC (Risk Assessment Committee of the European Chemicals Agency, ECHA) recommendations and, with the exception of lead and its compounds, none of them has been included in CAD since only BBLVs are recognised in this directive. The same applies to BGVs.

Therefore, member states are not obliged to set national BLVs/BGVs for the substances not included in CAD, Göen et al. [32] present an overview of biological values in different countries that are set to regulate internal exposure. This paper demonstrates that there are large differences between the limit values with regard to the underlying principle (health based, partially or not etc.). This fact helps explains the difficulties in recognizing and understanding the meaning of the different limit values adopted in the different countries.

Additionally, REACH regulation (European Regulation on Registration, Evaluation, Authorisation and Restriction of Chemicals) also allows the setting of derived no-effect levels (DNELs) for biomarkers, although it does not give much advice for their derivation. In contrast, the REACH IT-system (IUCLID) does not include a placeholder for internal biomarker-DNELs [7,33,34]. Although it was proposed more than 10 years ago that DNELs could serve as the basis to derive a biological equivalent/internal DNEL/biological guidance value (by applying the same methodology as was used to derive biological equivalents from other external reference values [35,36]), there are not many biomarker-DNELs currently available.

### 3.2. Skin Notation and Biomonitoring

Skin absorption, an exposure route common for substances used in occupational settings, has been highlighted in the OSH regulation through the use of the “skin notation” term. As mentioned in the Directive 2000/39/EC of 8 June 2000, *a skin notation assigned to the OEL identifies the possibility of significant uptake through the skin*. This definition is also mentioned in the Methodology for the Derivation of Occupational Exposure Limits defined by SCOEL [28]. In these cases, SCOEL recommended a skin notation when it can be assumed that dermal exposure may contribute to about 10% or more of the body burden by inhalation exposure at the OEL [28]. In 2017, a joint task force (RAC and SCOEL) reported that, in the case of dermally absorbed chemicals, biomonitoring (if available) would be a key component for the assessment of exposure [29]. Additionally, in 2019, ECHA also published guidance for preparing a scientific report for health-based exposure limits at the workplace. In this document, besides the previous message, it is also mentioned that biomonitoring generally allowed exposure from all sources to be assessed [37].

The American Conference of Governmental Industrial Hygienists also define ‘skin notation’ as “the potential significant contribution to the overall exposure by the cutaneous route” and state that “biological monitoring should be considered to determine the relative contribution to the total dose from exposure via the dermal route” and “the use of a skin notation is intended to alert the reader that air sampling alone is insufficient to quantify exposure accurately” [16].

Table 2 presents a list of all the EU OSH Legislation that mentions skin *as a possible exposure route to chemicals in the workplace* or uses the *skin notation* for specific chemicals. In this last case, the most common text associated in the legal text is “A skin notation assigned to the occupational exposure limit value indicates the possibility of significant uptake through the skin”. In the Directive 2004/37/EC, the text is different but the meaning is similar “Substantial contribution to the total body burden via dermal exposure possible”.

The high number of substances having a skin notation emphasises BM as a relevant exposure assessment tool. For substances that have the capacity to be absorbed by the skin special attention should be given to identify RMMs that can prevent skin contact, in addition to any inhalation RMMs.

### 3.3. Historical and Current Status of Biomonitoring in Occupational Safety and Health Regulations

**Figure 1:**
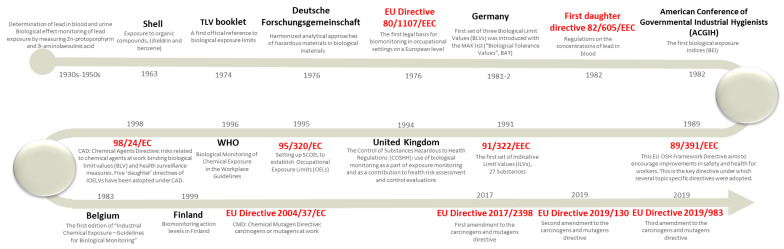
Sequence of steps towards an official implementation of biomonitoring (BM) in Europe.

The time sequence of steps towards an official implementation of BM in Europe is presented in Figure 1. 

BM in the occupational health setting was developed early in the 20th century with the determination of B-Pb [38] and benzene metabolites in urine of workers in different occupational settings [39,40]. Later, larger-scale BM was used in exposure and health surveillance programmes or as part of regulatory requirements, particularly for workers in the chemical industry [2]. Currently, BM of B-Pb is required by law in all EU countries. However, legal requirements for screening other chemicals will vary from country to country and this results in different BM applications and experiences, as is possible to observe in previous published reports [13]. Moreover, the most common reference to BM is made in the scope of health surveillance. This explains the requirements of each country concerning how BM data is stored (commonly as individual data in the worker’s clinical file, with the results being communicated individually to each worker) and, consequently, this prevents their use in exposure assessment. Indeed, in most of the regulatory areas (e.g., REACH), the data requirements for registration do not support the use of BM and it is only presented as an optional tool for exposure assessment [7]. Guidance on how to use BM in risk characterisation and management is very limited [41].

Therefore, better clarification of all the possible BM applications in the context of OSH such as: a relevant tool for exposure and risk assessment, to provide the information needed to define the RMMs to be implemented or to improve the ones already in place is of utmost importance.

## 4. Communication and Ethical Aspects to Consider When Using Biomonitoring Data in Occupational Safety and Health

Effective communication is essential for proper collection, interpretation and use of BM data in the occupational exposure assessment process. Without an effective communication process, undesirable outcomes may result. These can include anxiety, conflict, lack of confidence and trust, resulting in the study being viewed as a poor use of time and resources and the study outcomes being questioned. In the case of a BM research project being developed or a BM campaign taking place in a company site, communication between the field work team and the target worker population is key for the success of a BM campaign. The field work team need to have a clear understanding of the worker and company involvement required for the proposed study/BM campaign (e.g., type, number and timing of sample collection) and clearly communicate to the workers what is requested of them, the reasons why and the wider benefits of the study/BM campaign [17,42]. This dialogue might include asking the workers to define questions they want answered. Payne-Sturges et al. [43] noted that effective communication and translation of the exposure assessment approach enables the community to “credibly represent the study’s implications to policy makers and other stakeholders” Additionally, this initial contact also makes available information about workers’ preferences for results communication, both in terms of who provides it and dissemination method [42]. As an example, in the scope of the HBM4EU—science and policy for a healthy future (https://www.hbm4eu.eu/about-hbm4eu/) research project a BM study was developed aiming to collect new data on current occupational exposure to hexavalent chromium (Cr(VI)) in Europe, engaging eight countries. In this study it was decided that the study participants would receive their personal urinary chromium results from the research team or an occupational physician, depending on the country specific regulations (unless they have specifically indicated that they do not wish to receive feedback) [44].

Comparable to the general population, communication and dialogue with workers needs to be established in the initial phases of an exposure assessment process. This approach will result in several benefits, such as increased trust in those responsible for the BM campaign, increased use and relevance of the data obtained and improved dissemination [45,46].

In fact, one of the first steps that will influence the definition of to whom (and also how) the BM results will be communicated is to recognize the purpose of the BM campaign. If the main purpose is workers health surveillance, the data communication should be made by the occupational physician directly to each worker involved or, by someone that has been identified as the point of contact and dissemination during the informed consent, guaranteeing always the confidentially aspects; if the purpose is exposure and risk assessment, then the individual worker data should be aggregated in a way that guarantees data confidentiality and anonymization and, only in this case, can be communicated to the employer and, for instance, contribute to a more accurate exposure and risk assessment and, if needed, support the improvement of the risk management measures in place. However, in cases of individual results exceeding binding biological limit values (or even values that are higher than what is being seen at a group level), the occupational health team should analyze those individual results, considering the contextual data available, and act accordingly to prevent continued exposure and thereby ensuring the workers’ health protection. Actions can be focused on improving the risk-management measurements in place or, as a last resort or when other measures cannot be applied in a short period of time, to remove the worker from a specific workplace/task until exposure can be avoided or minimized. Nevertheless, the same BM data can be used for both purposes (health surveillance, exposure and risk assessment) resulting in a better use of resources. Additionally, they can also be used to inform and change workers’ behaviours to reduce or eliminate exposure. Bearing in mind all these possible applications of BM data, it is important to recognise the need for BM limit or guidance values. These provide, besides an easier interpretation of BM results, a clearer communication of results to workers and employers.

Considering ethical issues, Manno and co-authors summarized very well the main ethical aspects that should be considered when communicating BM data to workers [2]. They state that the content and the form of communicating the results to the workers, individually and collectively, should be decided and agreed upon early when planning the study. Attention should also be paid to ensure confidentiality and not to release data which might directly affect the worker(s) involved, his/her health or life insurance, or other sensitive personal issues [2]. Additionally, and given that close communities can exist in the working environment, it is important that those participating in the BM campaign within a given company receive results that have consistent and understandable messaging. To minimize potential anxiety, results to the participating workers should be provided in similar timeframes (with due consideration of collection timings) and that it is made clear what actions they and/or their company can undertake if remedial actions are considered necessary.

Considering all of the above issues, it is possible to recognize why it is important to define a communication plan, with enough resources and time, in the beginning of an occupational exposure assessment process that might implicate a BM campaign. This communication plan should present the details of what information to provide, to whom, when and in what format. For example, the communication plan should address the timely reporting of results to the workers that participated in the BM campaign and this is indeed a very relevant aspect to guarantee their willingness to participate in future BM campaigns. The communication plan should also explain how (and if) information will be given to the employers, community, the media, stakeholders, the scientific community and other groups as considered suitable by the research team and ethically appropriate by ethics committees [42]. However, and as mentioned before, this is very much dependent of the purposes of the BM campaign. A consensus has yet to appear concerning the ethics of reporting individual data on environmental exposures when the relationship between exposures and health outcomes is not established [47]. Indeed, some environmental health advocates and scientists who generally support the notion of community right-to-know remain wary of individual notification of data when the clinical implications are uncertain [48]. However, in a workplace scenario, it is possible to use BM for exposure assessment without necessarily understanding the health outcomes, using the basis that reduced exposure equates to reduced likelihood of ill-health and in line with the ‘as low as reasonably practicable’ approach often used for managing non-threshold chemical exposures (e.g., carcinogens).

At a company level, the purpose of the BM campaign should be identified in advance and a strategy to communicate individually the results to each worker should be decided (when, how and by whom). This should convey a clear message not only about the worker’s result but also the meaning of that result for their health risk. Moreover, the moment to communicate and explain the results should be used also for increasing workers’ health literacy through simple messages to allow workers to be more prepared to take the correct decisions to avoid exposure and to protect their own health. This communication should be performed by someone of the occupational health team since all these professionals observe the rules of confidentiality in the execution of their functions, as previously reported [49].

Bearing in mind what makes effective communication and given the central role of communication in the success of interpretation and use of BM data, developing an infrastructure should be of high priority for BM supporters and researchers. These efforts will also guarantee that the ethical aspects will be taken on board from the beginning of any BM campaign. In summary, to ensure success, effective communication and increase the success of interpretation and use of BM data, a number of actions should be developed. (1) Good and clear communication between the field work team and the target worker population from the beginning. (2) Explain the purpose and the wider benefits of the study/BM campaign to all concerned stakeholders. (3) Make efforts to adapt to workers communication style preferences whenever possible. (4) Ensure results confidentiality is at all times upheld. (5) Results need to be consistent, with clear actions being identified that workers and/or their company can undertake if remedial actions are considered necessary.

Without proper communication, the efforts of undertaking a BM campaign could be unsuccessful in fulfilling the most pertinent of all goals: protecting workers’ health.

## 5. Challenges for the Future

### 5.1. The Importance of Biomonitoring Databases

Various research institutes and commercial laboratories involved in BM are likely to maintain their own databases of BM data but these are generally not openly accessible as they will likely contain personal data. Some data have been published from these databases on an ad-hoc basis [50]. The IPChem (the Information Platform for Chemical Monitoring) database (https://ipchem.jrc.ec.europa.eu/) is starting to host BM data for the general population; this is mostly datasets uploaded from the HBM4EU project (https://www.hbm4eu.eu/).

These databases and others that should become available will facilitate standardisation and harmonisation of rapidly evolving components of exposure science and, consequently, risk assessment will benefit from well-designed BM studies. Furthermore, these databases will assist a national health authorities, EU agencies, policy-makers, the scientific community and civil society in coordinating research efforts and facilitating new approaches to communication on health risk assessment. Additionally, BM databases can be a good support for defining regulation needs and priorities as well as to evaluating the impact on exposure of regulatory actions already in place. This implies their continued updating with new data.

BM can also have an important role in the identification of new and emerging risks of chemicals. Although more commonly applied in general population studies, non-targeted analysis of chemicals can be (and is already) used for detection of both known and unknown chemicals in biological samples and is necessary to support further exploration of the total of occupational exposures [51]. It may help identify chemicals that are not currently considered for biological monitoring but need to be addressed [51,52,53]. Therefore, the availability of these innovative data in databases should be encouraged and supported.

### 5.2. Biomarkers of Effect and Susceptibility in Occupational Safety and Health

**Figure 2:**
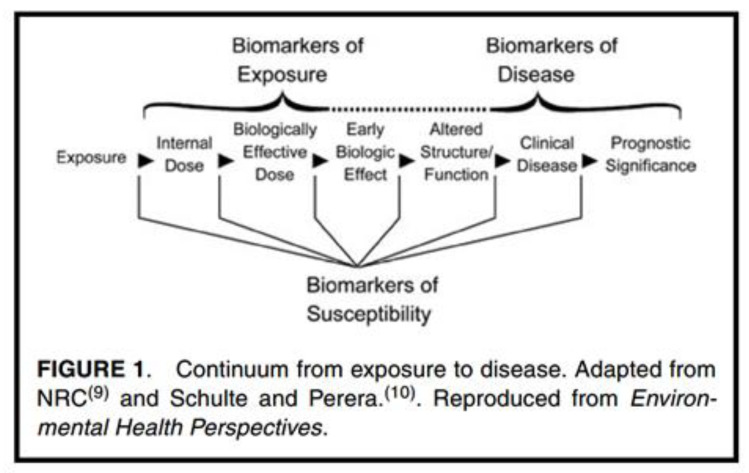
Continuum from exposure to disease. Source DeBord et al., 2015 [56].

The generally accepted classification of biomarkers divides them into three main categories: biomarkers of exposure, effect, and susceptibility [9,54,55]. As such, biomarkers fulfil a role in the continuum from exposure to effect and they can be used to inform (early) effects and to study the effects of an exposure or intervention (Figure 2).

Biomarkers of effect are (reversible) biochemical and functional alterations en route to the ultimate health effect. These biomarkers may be an endogenous component, or a measure of the functional capacity, or some other marker of the state or balance of the body or organ system, as affected by the exposure. Commonly it is a pre-clinical marker of pathology and can be specific or non-specific [57,58]. Specific biomarkers are useful because they indicate a biological effect of a particular exposure, thus providing evidence that can potentially be used in exposure prevention. The non-specific biomarkers do not point to an individual cause of the effect, but they may reflect the total, integrated effect due, for instance, exposure to a mixture. Some early effect biomarkers have essentially been used as ‘exposure biomarkers’ in OSH e.g., cholinesterase activity for organophosphate pesticides [59], carboxyhaemoglobin for carbon monoxide [60]—these tend to be less generic markers so whilst still not specific they are focused on a group of chemicals with a common adverse effect. Both types of biomarkers of effect are useful indicators of early (critical) effects and can signal higher risks for a particular group of workers, helping to define priorities for preventive actions [58]. Additionally, it is important to mention that effect biomarkers are the only tools that are able to address, in parallel, known and unknown risks of measurable and not measurable workplace relevant substances [61]. Thus, if there is a sufficiently established relationship between levels of effect biomarkers and clinical outcome (adverse effects), it is even possible to set a ‘background level’ of an internal effect biomarker. Once exceeded, it would trigger further research towards exposure to certain (mixtures of) chemicals or immediate exposure mitigation.

Therefore, effect biomarkers used as early predictors of clinical disease can advance occupational health risk assessment and trigger new effective disease prevention actions in occupational settings, but further validation is needed [2,26]. The potential of using effect biomarkers was also recognized recently by the OECD (Organisation for Economic Co-operation and Development) occupational biomonitoring activity of Working Party on Hazard and Exposure assessment and within the ISES Europe Human Biomonitoring working group and it is the main subject of a submitted publication (Zare Jeddi et al., submitted).

Tiered approaches need to be set up for establishing useful effect biomarkers, generating data on effect biomarkers and use of the results in (mixtures) exposure assessment. In the future, it may also be possible to perform preliminary mixtures exposure assessment by using multifactorial statistical correlation analyses to establish relationships between levels of various analytes and one effect biomarker. Another important research need is whether the presumed specific occupational biomarker can be raised by exposures outside the work e.g., by specific foodstuffs, hobbies, use of consumer products or environmental pollution. To address this it might be relevant to include a control group of the general population in order to exclude the possibility of the observed effect being related to other exposure sources and not the workplace environment. This is a common approach followed even when studying exposure (and not effect of) to contaminants that, besides being present in the workplace, are also food contaminants [62,63].

Concerning biomarkers of susceptibility, data may be useful in risk assessment and risk management efforts. However, it is important to mention that these are still very much under scientific development and they should be carefully evaluated in terms of sensitivity and specificity, interpretation, communication and management of the results, as well as from an ethical perspective to not discriminate or reduce job opportunities and the quality of life for involved workers [23,64].

Phenotyping regarding xenobiotic metabolising enzymes is possible, albeit not common. Rapid or slow metabolizers might be at increased health risks, dependent on whether metabolism exhibiting genetic polymorphism is activating or deactivating the parent substance. Phenotyping is not frequent and mostly used in hospitals for optimizing dose levels of human pharmaceuticals. If one wants to take this into account in occupational settings as well, transformation of current clinical tests is probably necessary (faster, cheaper, less invasive). Moreover, these biomarkers need to be specific for the chemical substance the worker is exposed to. Additionally, the co-exposure with medicinal drugs might be relevant as well because some of them can induce or inhibit xenobiotic biotransformation (e.g., cytochrome P450), thereby increasing the susceptibility to some occupational exposures [26].

### 5.3. Regulatory Needs to Improve the Biomonitoring Use in Occupational Risk Assessment

Under EU OSH legislation, the EU SCOEL has recommended biological limit or biological guidance values for more than 20 substances. These have not, however, been incorporated into the Chemical Agents Directive or Carcinogens and Mutagens Directive (CMD) except for B-Pb for which a BOELV has been established at EU level in the CAD. Therefore, except for B-Pb it is currently up to the member states how they apply SCOEL—recommended BLVs and BGVs in the exposure and health risk assessment of workers (https://echa.europa.eu/oel). This is the main reason for the variable use of BM in different member states. According to the paper by Louro et al., [7], legislation and the requirement for medical surveillance is one of the main drivers to perform BM in occupational settings. In some EU member states, because there is a BBLV (and obligation for BM) only for lead, BM is used only for B-Pb and not proposed for other chemicals. In addition to binding biological limit values, there should be also a possibility to give indicative BM limit values under CAD. Currently, there is no possibility for indicative BLVs in CAD or CMD. So far, indicative values have been published in European Commission Directives (external IOELV) and in SCOEL Opinions (BLV). See Table 2 for overview. The difference between binding and indicative values is that binding value may take into account socio-economic factors and in that sense they are not purely “health-data based”. In addition, for substances with a binding BLV, Member States must set a national biological limit value in their national legislation and that concentration should be equal or lower than the binding BLV. We suggest also setting indicative BLVs, as per IOELVs. They should be purely “health-data based” and Member States should set a national limit value in their national legislation. The indicative BLVs are a proposal for a national limit value but may be also higher. As explained earlier, BGVs are only statistically derived values, which only help to identify occupationally exposed from non-exposed workers and do not have any regulatory status (and do not need such).

A welcome recent development is the adoption of the 3rd wave amendment of the CMD. This includes an airborne binding OEL for cadmium and its inorganic compounds. It recognises the potential benefits of BM for this group of carcinogens and requests the Commission to assess the possibility of a BLV and to issue practical guidelines for BM.

As mentioned before, in the OSH framework (in CAD and CMD), BM is noted only as a health surveillance tool and not as an exposure assessment tool. As a consequence, BM is considered as medical data limiting the use of BM data in risk management at the workplace because of the privacy issues. This clearly needs regulatory clarification, since in most cases in practice, BM is measuring only exposure biomarkers and thus can inform only on exposure, and not on health (a clear individual relationship between internal exposure levels and health effects often has not been established). Thus, under the OSH framework, there is a clear need to explain the role of BM as an exposure assessment tool versus health surveillance tool.

Another important regulatory use of occupational BM data is in the context of REACH regulation. REACH includes a possibility to set DNELs for exposure biomarkers (biomonitoring or internal DNELs) in the chemical safety reports as prepared by industry or by RAC in their opinions. But in practice, these include limited methodological information on how they are derived and how they should be used in practice, and what the underlying uncertainties have been. In addition, as noted by Louro et al. [7], the REACH IT-system (IUCLID) does not include a placeholder for biomarker-DNELs. Where REACH exposure scenarios are in many cases based on modelled data, BM could have an important role in post-marketing surveillance [65] and its role here needs to be further clarified and strengthened. Currently, an OECD project on BM is aiming to create guidance for the derivation of biomarker-DNELs, which may help in increasing the use of BM. On the other hand, there are already some good examples of how BM data have been used in REACH restrictions and authorizations [7]. These include e.g., authorization application of MOCA [66]. The main obstacle for the use of BM data (even though they are available in companies) in the case of authorizations, is the privacy concern which some companies have claimed to prevent the delivery of even anonymized or aggregated (e.g., median values or P50 or P95) BM data for authorization purposes. There may be a need for further guidance on how to handle such data, although ECHA has already provided a template for the collection and delivery of aggregated BM data for authorization applications (https://echa.europa.eu/documents/10162/22979809/tmpl_reporting_occupational_exp_data_du_en.xlsx/84ef3203-4294-75c8-3b79-9c024abc2bcd).

Also in the case of pesticides (plant protection products) regulation, BM could have a more visible role along the external exposure monitoring as a tool to characterize exposure. According to a recent publication of EFSA (European Food Safety Authority) [67], available BM information could have a role in the validation and adjustment of parameter assumptions of exposure assessments models and in targeting new internal exposure measurements (choosing the best sampling time, e.g., before or after work or at the end of the working week) that should be required for the re-approval process. One aspect that may limit the use of BM in the pesticides regulatory arena is that the use of volunteer studies in the approval of new substances has not been allowed. Volunteer studies may be needed e.g., to identify new biomarkers and to study toxicokinetics (needed to design the measurement strategy). There is currently no EU regulation for post-approval monitoring but, like in the case of REACH, BM could have a big role in this post-market surveillance as suggested recently by the chief scientific advisors of the European Commission [68]. Collection of BM data should be added as a routine component of existing occupational health surveillance programmes for pesticide workers as a part of this post-market surveillance. Obtained BM values could be used for validation and amending previously assumed and used exposure model parameters.

### 5.4. Needs for Guidance and Education

There is a need for guidance and education of risk assessment and OSH experts at different levels. First of all, there is a need for clear guidance and best practices for regulators for the setting of biological limit and guidance values. Under OSH, SCOEL had guidelines for this but RAC has taken over SCOEL’s tasks, and RAC refers to existing practices of SCOEL and e.g., the French Agency ANSES (Agence nationale de sécurité sanitaire de l’alimentation, de l’environnement et du travail) and the German Commission for the Investigation of Health Hazards of Chemical Compounds in the Work Area (MAK Commission) OSH agencies but does not have any detailed guidance of their own [38]. Also detailed guidance on how to set DNELs for biomarkers for RAC in their opinions and for industry in their Chemical Safety Reports is missing. The ongoing OECD biomonitoring project might help in this since it aims to produce guidance for the setting of health-based limit values and guidance values for occupational exposure (in general for regulators as well as for industry) and could provide an international standard for this.

Importantly, the mere setting of biological limit/guidance values or DNELs is not enough in order to ensure the correct use of BM. This needs both general guidance on the use of BM in occupational settings as well as substance specific guidance.

General guidance should include guidance on the setting-up of the BM campaign at the workplace and recruitment of workers, collaboration between the occupational physician and industrial hygienist to set up the study and to interpret the results at the group level. Furthermore, it might be that the use purpose (exposure assessment or health surveillance) necessitates some specificity in the generation and interpretation of the BM data. Therefore, clarification of the purpose of the actual BM should be included, i.e., as an exposure assessment tool versus a health surveillance tool. Guidelines for dealing with confidentiality and General Data Protection Regulation issues in the case of medical data are also needed. The legal basis of BLVs and BGVs needs to be explained as well as the difference between BBLVs in the CAD (and maybe in future in the CMD) and (possible) indicative biological values that have been published only in SCOEL Opinions (see also Table 2). General rules for the actions to be taken in the case of exceedance of BGVs/BLVs (actions at the workplace vs. actions targeted to individual workers)—what actions should/could be taken, what shall not be done as previously mentioned when communicating and acting on BM results. In addition, for every single substance for which there will be a BLV or BGV, there is a need for a substance specific guidance covering several aspects. Check-lists both on pre-analytical requirements and analytical conditions are available and should be complied with [69]. Substance specific information should be available regarding:

- legal status of the biological limit value (binding or indicative);
- substance specific sampling strategy (including specific sampling time considering toxicokinetic data);
- possibilities for pre-analytical errors (contamination issues, wrong sampling time etc.), other exposures which might have an impact on the results (medicines, tobacco smoking, food);
- analytical requirements, quality assurance;
- interpretation of results; what does it mean if the measured value exceeds BGV/BLV, what actions should/should not be taken;

Moreover, a separate guidance is probably needed for the use of BM in REACH/plant protection products contexts, explaining how BM is used in post-market surveillance, what the biomarker-DNEL means, what are the (legal) consequences if it is exceeded etc.

### 5.5. Needs for Future Research to Support the Use of Biomonitoring in Occupational Risk Assessment

To achieve wider acceptance of BM as an exposure assessment tool, greater obligation to perform post-marketing surveillance occupational BM is needed. With this obligation, there are also research needs to make the regulatory obligation work and for it to be meaningful.

Regulatory obligations in the near future will likely trigger further research. Early research results will enable more concrete law-making.

As health risk is driven by exposure, the exposure–effect relationship and susceptibility, there is a need to address all three related types of biomarkers in research needs, at least for part of the tens of thousands of chemical substances that are commercially available. For all three, regulatory needs already exist or are expected in the future. Right now, there are only obligations (mainly health surveillance though) for exposure biomarkers, and none for effect and susceptibility biomarkers. However, with increased knowledge and use cases as well as regulatory need, such as e.g., for combined exposure to substances with similar modes of action, this is expected to change. Exposure to mixtures, the most common scenario concerning exposure in the workplaces, can likely be assessed and any identified risks effectively controlled with sufficient knowledge and use of effect biomarkers.

One central need, without which sensible use of BM data in an occupational health and safety context is not possible, is a thorough understanding of the relationship between external and internal exposure. Internal exposure is the best exposure measure, but risk mitigation measures can only be taken at the external exposure level. And as the relationship between external exposure (e.g., ambient air monitoring or personal air sampling) and internal exposure (exposure biomarker) is time-dependent, it is obvious that knowledge of the kinetics of a substance following exposure via inhalation or dermal exposure is of utmost importance, i.e., in order to know what to measure with the BM tool, one needs to know the most suitable exposure biomarker, be it the parent compound, a metabolite or the sum of both. For this, thorough knowledge on the parent, its metabolites and their appearance over time is needed.

Therefore, and focusing on exposure biomarkers, there is a need for more and better human data regarding absorption, distribution, metabolism and excretion. Also needed are blood concentration-time curves and urinary (and sometimes respiratory) excretion profiles (which is toxicokinetics) for each occupational exposure relevant chemical substance. Crucial is knowledge on absorption rates, distribution (including whole blood vs serum vs plasma), metabolism (qualitative and quantitative) and excretion (routes and rates) of workplace-relevant substances and under worker-relevant circumstances.

Sometimes the above needs to obtain sufficient knowledge on the toxicokinetics of a substance can be addressed by carefully studying actual occupational exposure scenarios. However, there may be limitations to what is possible in order to delineate toxicokinetic information from real-life situations when accurate external exposure levels are needed. This might refer e.g., to substances for which peak exposures via inhalation are relevant for possible effects and where the relevant half-life is short and non-frequent sampling will not sufficiently reflect exposure. It might also refer to actual dermal exposure in terms of duration (hours or minutes), surface area concentration (µg/cm^2^), surface exposed (cm^2^), partial occlusion by personal protection equipment such as protective clothing or respiratory protective equipment. This could make it imperative to use human volunteer studies, e.g., mimicking actual daily occupational exposure under laboratory conditions, as long as they are performed by strictly following ethical guidelines.

## 6. Conclusions

BM is a highly relevant tool for occupational exposure and risk assessment. It is able to integrate exposure from all routes and can help identify unintentional and unexpected exposures. It can provide information on the effectiveness of existing RMMs (and the potential need for more) and the suitability of personal protective equipment together with an individual worker exposure assessment. Detailed BM data can also be used in conjunction with health data in occupational epidemiological studies. Furthermore, BM can provide relevant information to support policy making by delivering better evidence of the exposure of workers to chemical substances, allowing for the evaluation of the impact of regulations and the need for new or improved OSH regulation.

However, despite these benefits, BM is currently an underused exposure assessment tool. Several actions have been proposed to maximise the benefits and opportunities that this exposure assessment tool presents. Firstly, efforts for the legal enforcement of biological limit values in EU should be advanced. Secondly, clarity should be given (through regulation and guidance) on the valuable role that BM can have in workplace exposure assessment and risk assessment/management, as well as workers’ health surveillance. Thirdly, clear guidance should also be created for supporting regulators that have the responsibilities of setting biological limit and guidance values. In addition, regulators should communicate to the research community where biological limit and guidance values for a substance were not possible due to lack of data. General and substance specific guidance is also needed for occupational physicians and industrial hygienists on the management of BM campaigns and how to deal with ethical and General Data Protection Regulation issues.

Finally, it will be important to develop guidance to help employers set up a BM programme as part of their overall approach to chemical risk management at the workplace. This should include information for workers who participate in such programmes and should explain the purpose of BM, how it will be carried out, and how the results will be used.

These actions will help understanding the approach used to define limit and guidance values, to understand their utility for exposure assessment and health surveillance, to identify the needs for further research and to increase the use of BM by occupational health professionals.

## Figures and Tables

**Table 1:** Occupational limit and guidance values for external (airborne) and internal exposure.

	Binding	Indicative	
	Health-and Socio-Economic Factors Based ^1^	Health-Based	Statistically-Derived Guidance Value ^2^
External	Binding Occupational Exposure Limit Value (BOELV) In: CAD and CMD ^2^	Indicative Occupational Exposure Limit Value (IOELV) In: Commission Directives ^3^	--
Internal	Binding Biological Limit Value (BBLV) (not necessarily health based)	Biological Limit Value (BLV) (health based threshold values)	Biological Guidance Value (BGV) (not necessarily health based; reference value for background exposure)
	In: CAD	In: SCOEL Opinions	In: SCOEL opinions (e.g., MOCA, MDA, Nickel)
Member States ^4^	National level to be set ≤BOELV or BBLV	National level taking into account the EU-level indicative value	

CAD: Chemical Agents Directive; CMD: Carcinogenic and Mutagenic Directive; “Indicative” means that there are health-based limits conventionally established only for substances for which it is possible to establish a threshold or a no effect level considered to be protective of health; “Binding” means that a value may be drawn up at a Community level on the basis of evaluation socio-economic and technical feasibility factors and on the availability of measurement techniques. Member states are obliged to set a national value that is lower or equal to the binding value. ^1^ https://oshwiki.eu/wiki/Occupational_exposure_limit_values. Examples: asbestos (several silicate minerals), benzene, hardwood dusts, lead (plus inorganic compounds), vinyl chloride monomer. ^2^ https://oshwiki.eu/wiki/Biological_monitoring_(biomonitoring). ^3^ https://echa.europa.eu/oel. ^4^ Member States are obliged to set national limit values for which EU-wide binding or indicative limits have been published in the Official Journal of the EU.

**Table 2:** List of European Union (EU) occupational safety and health (OSH) legislation that mentions skin notation.

EU OSH Legislation	Exposure Route	Skin Notation	No. of Subs. with Skin Notation/No. of Subs. Listed
Directive 91/322/EEC—indicative limit values	**x**		
Directive 2000/39/EC—a first list of indicative occupational exposure limit values		**x**	25/63
Directive 2006/15/EC—a second list of indicative occupational exposure limit values		**x**	9/33
Directive 2009/161/EU—a third list of indicative occupational exposure limit values		**x**	8/19
Directive 2017/164/EU—a fourth list of indicative occupational exposure limit value		**x**	10/31
Directive 2019/1831—a fifth list of indicative occupational exposure limit value		**x**	3/10
Directive 2004/37/EC—risks related to carcinogens and mutagens at work		**x**	1/3
Directive 2017/2398—first amendment to the carcinogens and mutagens directive		**x**	5/14
Directive 2019/130—second amendment to the carcinogens and mutagens directive		**x**	12/22
Directive 2019/983—third amendment to the carcinogens and mutagens directive		**x**	1/5

## Abbreviations

ACGIH	American Conference of Governmental Industrial Hygienists
B-Pb	Blood Lead
BBLV	Binding Biological Limit Value
BGV	Biological guidance value
BLV	Biological limit value
BM	Biomonitoring
BOELV	Binding Occupational Exposure Limit Value
CAD	Chemical Agents Directive
CMD	Carcinogenic and Mutagenic Directive
DNEL	Derived No-Effect Level
ECHA	European Chemicals Agency
ESFA	European Food Safety Authority
IOELV	Indicative Occupational Exposure Limit Value
ISES	International Society for Exposure Science
IUCLID	International Uniform Chemical Information Database
NOAEL	No-Observed-Adverse-Effect-Level
OECD	Organisation for Economic Co-operation and Development
OEL	Occupational Exposure Limit
OSH	Occupational Safety and Health
PFAS	Perfluoroalkyl and Polyfluoroalkyl Substances
PPE	Personal Protective Equipment
RAC	Risk Assessment Committee, European Chemicals Agency
REACH	Registration, Evaluation, Authorisation and Restriction of Chemicals
RMM	Risk Management Measure
SCOEL	Scientific Committee on Occupational Exposure Limits
SEGs	Similar Exposure Groups
WHO	World Health Organisation
